# Joining Forces: Establishing a Cardio-oncology Clinic

**Published:** 2018-03-01

**Authors:** Caroline Austin-Mattison

**Affiliations:** Yale University School of Nursing, New Haven, Connecticut

## Abstract

Patients have been surviving cancer diagnoses at a steadily increasing rate over the past few decades. Despite the encouraging decline in cancer morbidity, the cardiovascular effect of some chemotherapy medications is concerning. Moreover, even though there is extensive knowledge of the pathophysiology and increased risk of cardiotoxicity, there is a lack of specific guidelines and adequate cardio-oncology programs focused on reducing cardiovascular risks or disease in patients undergoing cancer treatment. The high incidence of both cardiovascular disease (CVD) and cancer warrants the collaboration of oncology and cardiology providers to screen and promptly treat CVD, and thereby provide an opportunity to improve cancer patients’ quality of life both during treatment and extended through cancer survivorship. We found no designated cardio-oncology programs in our geographic area from Poughkeepsie to New York City; therefore, a feasibility project was developed in collaboration with a cardiologist and a cardiology nurse practitioner specializing in oncology at our institution. The project included the development and implementation of a cardio-oncology program in a suburban community cardiology practice in order to provide service in this area to breast cancer patients at risk for cardiotoxicity. The Reach, Effectiveness, Adoption, Implementation, and Maintenance (RE-AIM) framework was employed to guide the project and enhance the translation of research into practice through planning and execution.

The survival rate of patients with cancer has increased over the past 25 years in the United States. The 5-year relative survival rate of patients diagnosed with cancer between the years 1975 and 1977 was 49% for all cancer sites, and this number improved to 68% between 2004 and 2010. When assessed by ethnicity, a 69% improvement in Caucasians and a 62% improvement in African Americans were noted for cancer of all sites. Breast cancer has a relative survival rate of 92% in Caucasians and 80% in African Americans (American Cancer Society, 2015). However, with increased survival rates comes an increased risk of cardiotoxicity.

Five percent of cancer patients develop heart failure, with the threat found to be higher in those patients with cardiovascular risk factors such as hypertension (HTN) and hyperglycemia. In a study by Hooning and colleagues (2007), women with early-stage breast cancer had a 30% increased risk of cardiac events, of which congestive heart failure was the most common. There are over 62.9 excess cardiac events in patients with breast cancer per 10,000 patient-years (Khouri et al., 2012). However, control of these cardiovascular risks and early heart failure treatment can result in complete recovery (Cardinale et al., 2010).

The increased incidence of cardiovascular disease (CVD) is a result of the adverse effects of chemotherapy agents. Patients treated with chemotherapy, especially anthracyclines, are prone to increased cardiovascular risk and may develop cardiomyopathy, heart failure, or myocardial infarction up to 20 years after treatment (Cardinale, Bacchiani, Beggiato, Colombo, & Cipolla, 2013). The role of anthracyclines in breast cancer treatment is continuing to expand due to its efficacy and tumor response, especially when used with antitumor medications such as taxanes and trastuzumab (Herceptin; Pouillart, 2004); therefore, it is important to assess for CVD and closely monitor patients at increased risk so they can receive appropriate cancer treatment without the increased risk of cardiotoxicity. The focus can no longer be solely on eradicating cancer without concern for the long-term effects of CVD. Because cardiac toxicity can occur at the start of chemotherapy administration, as well as months or years later, the cardiology provider’s role is to work with the oncologist and monitor for signs of cardiotoxicity. The severity of cardiotoxicity depends on molecular sites of action, immediate or cumulative dose of chemotherapy, methods of administration, presence of other comorbidities, and demographics (Yeh et al., 2004). During the surveillance phase, if a patient shows signs of cardiac toxicity, prompt intervention is warranted.

This early intervention will be possible only when providers work collaboratively to provide cardio-oncology care. Cardio-oncology addresses the cardiovascular side effects of cancer therapy to improve the quality of life for cancer survivors. The need for this specialty continues to increase with improvement in cancer risk factor identification, early screening, detection, and the success of new cancer treatments. As such, it is necessary to establish a cardio-oncology program that ensures the comprehensive management of any comorbid cardiovascular issues a patient has, in addition to any other problems associated with the patient’s cancer in order to maintain optimal quality of life throughout survivorship.

## EVIDENCE FOR EARLY INTEGRATION OF CARDIO-ONCOLOGY CARE

There are currently no cardio-oncology–specific guidelines that address the screening or management of chemotherapy-induced cardiotoxicity (symptomatic or asymptomatic) or the progression of cardiotoxic left ventricular (LV) dysfunction. However, current evidence makes it clear that prompt intervention improves outcomes and quality of life following the use of chemotherapy. Currently, the American Heart Association and American College of Cardiology (AHA/ACC) 2016 guidelines for the management of heart failure are being used to treat chemotherapy-induced cardiotoxicity (Yancy et al., 2016). The European Society for Medical Oncology did develop clinical practice guidelines in 2012 specifically for cancer patients, but those guidelines were based mostly on consensus. No randomized control trials were used to determine approaches (Cardoso et al., 2014).

**Figure 1:**
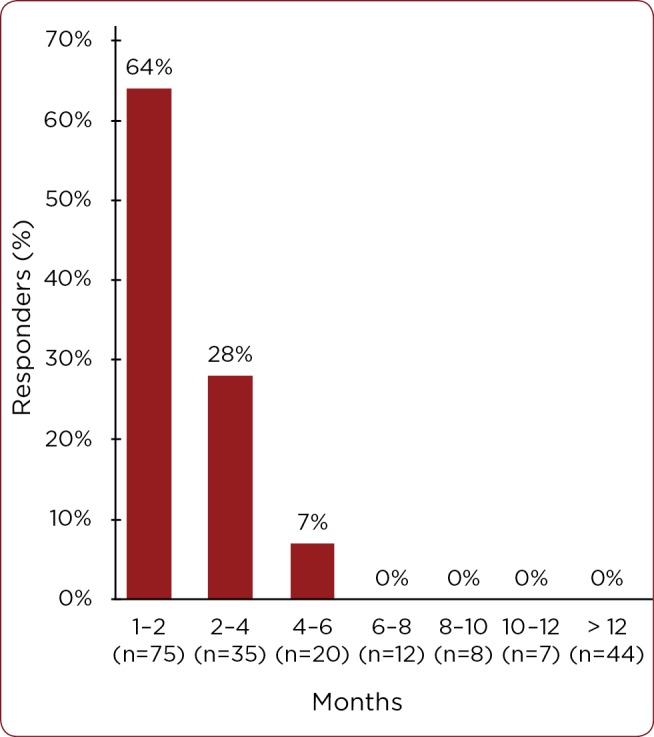
Percentage of responders according to the time elapsed from anthracycline administration and start of heart failure therapy. Permission granted from Daniela Cardinale, European Institute of Oncology, 12/2015.

Despite a lack of standardized approaches, there is evidence that the early treatment of patients who develop anthracycline chemotherapy-induced cardiotoxicity may result in the full recovery of LV function with improved cardiac outcomes (Cardinale et al., 2010). Figure 1 demonstrates a possible fourfold decrease in LV recovery based on how long it takes for treatment to be initiated after chemotherapy has ended. Of the patients who were treated within 2 months of the end of chemotherapy, 64% had full recovery of LV function. For every 2 months that treatment was delayed thereafter, the chance of full recovery decreased significantly (Cardinale et al., 2010).

**Figure 2:**
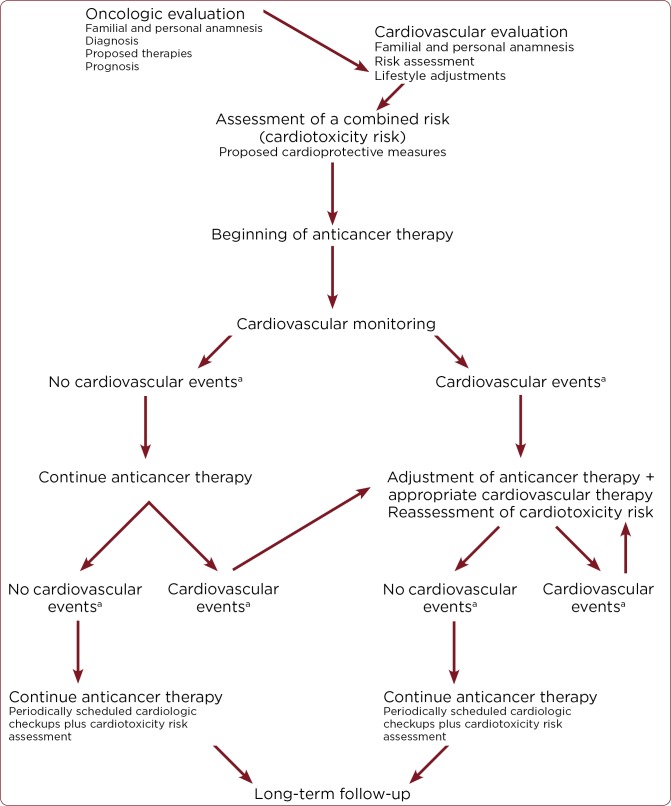
Cardiovascular monitoring of cancer patients. Adapted from Albini et al. (2010). Approval for use from Adriana, Albini; permission from Oxford University Press. a Substantial changes in cardiovascular risk assessment; for example, a reduction in left ventricular ejection fraction (LVEF) from baseline greater than 5% to less than 55% with accompanying signs or symptoms of HF or a reduction in LVEF greater than 10% to less than 55%, without accompanying signs or symptoms.

As such, it is clear that the optimal timing of heart failure diagnostic evaluation, selection of chemotherapy agent based on cardiovascular comorbidities, and utilization of cardio-protective regimens may result in longer survival and better quality of life (Albini et al., 2010). Patients who develop cancer and cardiac disease demonstrate varied outcomes depending on which specialty provider they see first. For example, if the patient sees the oncologist first, then cardiovascular risk factors might not be assessed and underlying cardiac disease may be missed (Albini et al., 2010). The risk of heart disease superimposed on a cancer diagnosis acts as a compelling argument for collaborative practice between the oncologist and the cardiologist early in the treatment process (see Figure 2). The use of a "sliding door" approach, in which both providers consider risks and coordinate care based on patient age, prior use of anthracycline, uncontrolled hypertension, uncontrolled diabetes, and/or cardiac disease, is essential in order to improve outcomes for these kinds of comorbidities (Albini et al., 2010).

## BENEFITS OF A CARDIO-ONCOLOGY CLINIC

The American Heart Association recommends close monitoring of cardiac function for adults undergoing anthracycline therapy alone or in combination. Unfortunately, it fails to provide a specific guide as to how often patients need to be followed and which methods and thresholds should be used (Sawaya, Plana, & Scherrer-Crosbie, 2011). Therefore, cardio-oncology providers who focus on cancer survivorship should be accessible to all patients who are at risk for cardiotoxicities associated with chemotherapy.

## THE CREATION OF A CARDIO-ONCOLOGY PROGRAM

The need for a cardio-oncology program in the Mid-Hudson Valley region is indisputable given the number of local breast cancer survivors. In Dutchess County, the number of cases for all malignant tumors among females averaged 472.8 per 100,000 annually during the same period (New York State Cancer Registry, 2017). Despite the need for these services, a search of cardio-oncology clinics revealed none within a 100-mile radius of the Hudson Valley. Given the dearth of cardio-oncology programs in the area, this project aimed to bring to fruition a sustainable cardio-oncology program to benefit breast cancer survivors.

A needs assessment that consisted of reviewing the electronic health records of patients referred for cardiology evaluation after developing symptoms of heart failure, performed by a nurse practitioner, was the first step in identifying the lack of access to comprehensive cardiology care among breast cancer survivors. Patients reported that they were experiencing symptoms related to cardiotoxicity postchemotherapy. The oncologists and administrators readily supported the initial formation of a cardio-oncology clinic for women with breast cancer. The program that was created utilized a proactive approach to managing patients’ needs identified from the assessment. Aspiring to provide care for the whole patient, coordination, planning, and education of patients on cancer and cardiac diagnosis and treatment, was key to creating the program. The initiation and growth of the program was well supported by the administrators and the other cardiologists in the group. Dedicated time for community outreach and providing education for oncology providers was most challenging for the advanced practice registered nurse (APRN) and cardiologist in a busy cardiology practice.

**The RE-AIM Framework**

The RE-AIM framework was used to guide the project and enhance the translation of research into practice through planning and execution. The RE-AIM model was applied as a structure to improve consistency in cardio-oncology disease management based on existing research in various settings. The framework has also been used to translate research into practice and to assist with program evaluation. The five steps of the RE-AIM model are Reach, Effectiveness, Adoption, Implementation, and Maintenance (Glasgow, Vogt, & Boles, 1999). This project was designed to incorporate three of those steps: Reach, Adoption, and Implementation.

The first step of the framework, Reach, was accomplished through a process that involved reaching the patients so they could be assessed and evaluated for their risk status of CVD. This process included increasing oncology providers’ awareness of the importance of early cardiovascular screening and soliciting their referrals for consultation. The goal was to improve access and consistency while reaching the target population: patients diagnosed with breast cancer requiring cancer treatment and oncology services. The cardiologist had been practicing in his specialty and the Hudson Valley region for 3 years and the APRN had experience working in cardiology for 9 years. Both providers had a goal to increase patient referrals, pretreatment assessment, and cardiovascular treatment as needed during and after chemotherapy treatment. The number of patients with breast cancer over a 6-month period varied weekly; however, most patients had a diagnosis of breast cancer and others forms included lung cancer, liver adenocarcinoma, and one patient with a diagnosis of multiple myeloma.

The Adoption of this project occurred as providers focused on screening and treating CVD risks in a timely manner. It involved the growth of the cardio-oncology program and it increased as women were referred for screening for pre-oncology treatment. The Implementation of this essential cardio-oncology program became possible with stakeholder support from administration and the medical oncologists in establishing this designated program in the Mid-Hudson Valley area. Maintenance of the program requires an ongoing effort to continually increase and achieve precancer treatment screening and routine follow-up care. Table 1 provides examples demonstrating the application of the RE-AIM framework in developing this cardio-oncology program.

**Table 1:**
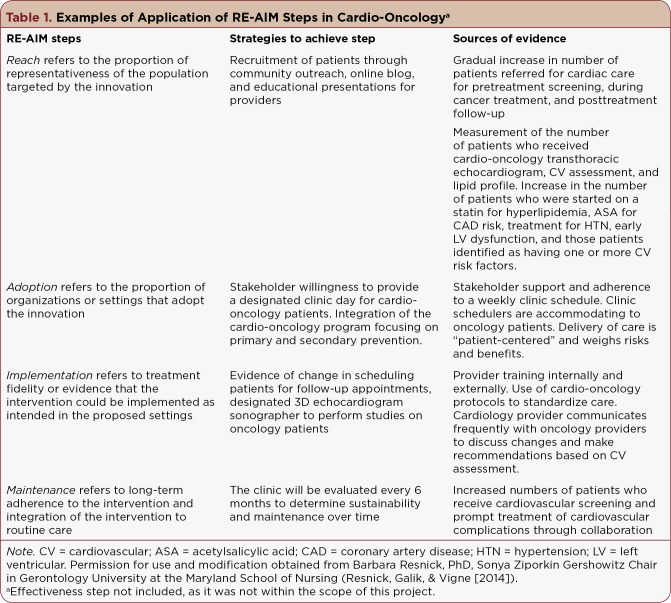
Examples of Application of RE-AIM Steps in Cardio-Oncology^a^

**Figure 3:**
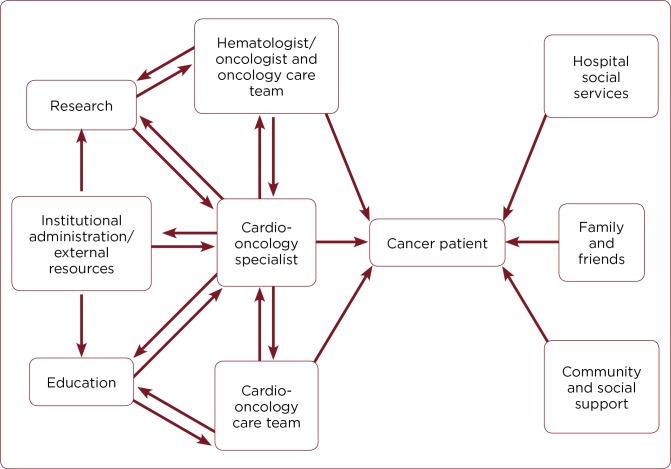
Cardio-oncology care team model demonstrating the interactive process between cardiooncologists, oncologists, patients, health-care administrators, and education required for integrated patient care. Permission granted for use by Dr. Okwuosa (Okwuosa & Barac [2015]).

A successful program requires ongoing interaction among cardio-oncologists, oncologists, nurses, and patients. The feasibility of developing and implementing this initial program was organized using the care team model (Figure 3), where integrated services were provided by a cardio-oncologist and an APRN. Developing the program involved orienting providers to the need for the program and teaching them about the diagnosis and management of cardio-oncology toxicities. Training was provided to oncologists, nurses, and support staff such as medical assistants, technicians, administrative staff, and leaders in the practice. Lectures were provided in various forums, such as an allied health provider education day, hospital symposia, and small group meetings. Meetings with leaders of the cardiology and community oncology practices were scheduled at regular intervals to increase awareness and maintain interest in the program.

A team effort leads to better-coordinated cardio-oncology appointments and diagnostic tests, and provides consistently timely scheduling and embedded time slots for patient education and engagement. Advanced practice registered nurse involvement in this program also improved clinical flow. The APRN saw stable patients during follow-up visits independently (scope of practice may vary based on institution or state policies) and began initial visit assessments and data collection. This approach provided the cardio-oncologists an opportunity to see more acutely ill patients.

**Outcomes**

Teaming the APRN with a local cardio-oncology provider for individualized cardiac risk assessment has proven to be beneficial. Under a collaborative care regimen, patients received individualized care that prevented further deterioration and led to improvement in cardiac function. The screening in this program was shown to not only help prevent chemotherapy-induced cardiotoxicity, but also prevent high-risk patients from receiving more toxic chemotherapeutic agents. Providers were informed and could use this information to offer choices based on risks vs. benefits, with an emphasis on improving disease prognosis and quality of life. The cardiology provider’s focus on reducing or controlling risk factors such as uncontrolled HTN, hyperlipidemia, and uncontrolled diabetes before and during treatment was a key element in this program. As a result, the program has been judged to be feasible and effective. Consequently, the cardio-oncology program is now a local service for Hudson Valley residents (see Figure 2 for the recommended cardiovascular monitoring algorithm).

**Barriers to Collaborative Care**

Although this project demonstrated that women with breast cancer were referred and assessed for CVD, there were some obstacles to increasing referrals and getting all oncology providers on board. This initial project was limited to women with breast cancer; therefore, a limited number of medical oncologists participated. Patients with other types of cancer scheduled for chemotherapy may be equally at risk for cardiotoxicities; as such, the referral network needs to be expanded to include other types of oncologists. As the program is expanded to patients with other cancers, additional efforts will be needed to obtain both institutional and provider buy-in. It is well recognized that a lack of institutional support may hinder the growth and success of any cardio-oncology program (Okwuosa & Barac, 2015). There is also a need to continue to develop evidence-based standards because there is little consensus on a standard of practice (Okwuosa & Barac, 2015). There is also a lack of clinical trials demonstrating outcomes associated with cardio-oncology management, which may limit the oncologist’s willingness to refer to a cardiologist. Referring patients with two or more risk factors may not seem to be a priority for some providers; however, pre-existing HTN and diabetes should be concerning in women undergoing chemotherapy, as the Framingham Heart Study general cardiovascular risk profile establishes HTN, smoking, dyslipidemia, and diabetes as major CVD risk factors (D’Agostino et al., 2008). Despite the barriers encountered during the development of the project, working with the targeted population and applying a commonly used care model was valuable in its implementation.

## DISCUSSION

It is well established that the number of cancer survivors is increasing. Therefore, ensuring the best quality of life for patients following a cancer diagnosis and treatment is important. Because many patients treated for cancer fear recurrence and are concerned with their long-term wellness, there is an opportunity for cardio-oncology providers to impact survivorship. Providers interested in decreasing cardiac events and promoting wellness among cancer survivors need to shift their focus to include early screening and treatment. Cardio-oncology care should not be restricted to those who have already developed cardiac complications. The cardio-oncology program described here is not only closing the gap between cardiovascular assessment, prevention, and prompt treatment, but also providing a necessary local service for improving the wellness and quality of life of patients after their breast cancer treatment. This data can be useful for any advanced practice provider who aims to improve the quality of life for cancer survivors. Prompt monitoring and intervention for patients undergoing oncologic treatment for breast cancer should be the goal of all providers.

**Acknowledgment**

I would like to express the deepest appreciation to my committee chair, Dr. Jessica Coviello, who introduced me to cardio-oncology and whose enthusiasm to improve the quality of life for cancer survivors sparked my interest. Dr. Coviello consistently challenged me and supported my development and the fruition of this scholarly project.

I would like to give special thanks to Dr. Ruth McCorkle at Yale University School of Nursing, Dr. Kamran Haleem at the Hudson Valley Heart Center, and Dr. Gagan Sahni at Mount Sinai Medical Center for each of their expertise and contribution to this project. This Capstone project could not have been successful without the assistance, encouragement, and support of these brilliant professors and clinicians, who actively participated and guided my project. This support afforded me the opportunity to translate evidence-based care into practice and improve systems of care and outcomes of cancer patients in the community.
